# Association of a single nucleotide polymorphism in *akirin 2 *gene with marbling in Japanese Black beef cattle

**DOI:** 10.1186/1756-0500-2-131

**Published:** 2009-07-14

**Authors:** Seiki Sasaki, Takahisa Yamada, Shin Sukegawa, Takeshi Miyake, Tatsuo Fujita, Mitsuo Morita, Takeshi Ohta, Youichi Takahagi, Hiroshi Murakami, Fumiki Morimatsu, Yoshiyuki Sasaki

**Affiliations:** 1 Maebashi Institute of Animal Science, Livestock Improvement Association of Japan, Maebashi, Gunma 371-0121, Japan; 2 Laboratory of Animal Breeding and Genetics, Graduate School of Agriculture, Kyoto University, Sakyo-ku, Kyoto 606-8502, Japan; 3 Research and Development Center, Nippon Meat Packers, Inc., Tsukuba, Ibaraki 300-2646, Japan; 4 Cattle Embryo Transfer & Genetics Section, Oita Prefectural Institute of Animal Industry, Takeda, Oita 878-0201, Japan; 5 Kyoto Cattle Genetics Section, Beef Information & Genetics Institute, Inc., Otsu, Shiga 520-0865, Japan

## Abstract

**Background:**

Marbling defined by the amount and distribution of intramuscular fat, so-called *Shimofuri*, is an economically important trait of beef cattle in Japan. The c17-25 expressed sequence tag (EST) has been previously shown to possess expression difference in *musculus longissimus *muscle between low-marbled and high-marbled steer groups, and to be located within genomic region of a quantitative trait locus for marbling. Thus, the *akirin 2 *(*AKIRIN2*) gene containing the c17-25 EST sequence was considered as a positional functional candidate for the gene responsible for marbling. In this study, we explored single nucleotide polymorphism (SNP) in the *AKIRIN2 *and analyzed association of the SNP with marbling.

**Findings:**

A SNP in the 3' untranslated region of the *AKIRIN2*, referred to as c.*188G>A, was the only difference detected between high- and low-marbled steer groups. The SNP was associated with marbling in 3 experiments using 100 sires (*P *= 0.041), 753 paternal half-sib progeny steers from 4 sires heterozygous for the c.*188G>A (*P *= 0.005), and 730 paternal half-sib progeny steers from 3 sires homozygous for the *A *allele at the c.*188G>A (*P *= 0.047), in Japanese Black beef cattle. The effect of genotypes of the SNP on subcutaneous fat thickness was not statistically significant (*P *> 0.05).

**Conclusion:**

These findings suggest that the *AKIRIN2 *SNP polymorphism is associated with marbling and may be useful for effective marker-assisted selection to increase the levels of marbling in Japanese Black beef cattle.

## Background

Generally, marbling means the amount of intramuscular fat [1]. In Japan, marbling is characterized as the amount and distribution of intramuscular fat in a cross section of *musculus longissimus *muscle, and called *Shimofuri *[1]. High levels of such marbling improve the palatability and acceptability of beef by affecting the taste and tenderness of the meat [2-4]. Because of the importance of marbling on the economics of beef production, there is great interest in gaining a better understanding of the molecular architecture of marbling and in generating new opportunities for more effective marker-assisted breeding.

We have previously undertaken differential-display PCR (ddPCR) in low-marbled and high-marbled steer groups at 8, 10, 12, and 14 months of age, encompassing the time that marbling starts to appear, to explore genes showing marbling-associated expression changes in *musculus longissimus *muscle [5]. Among the detected genes, the c17-25 expressed sequence tag (EST) showed higher expression levels in low-marbled steer group than in high-marbled steer group in the middle and late stages of the test period [5]. We have also located the c17-25 EST within genomic region of a quantitative trait locus (QTL) for marbling [6], which is mapped in a half-sib family of Japanese Black cattle to bovine chromosome 9 region [7]. The c17-25 EST sequence corresponds to a portion of the *akirin 2 *(*AKIRIN2*) gene. Thus, the *AKIRIN2 *gene was regarded as a positional functional candidate for the gene responsible for marbling.

### *AKIRIN2 *gene structure

We screened the NCBI bovine genome sequence database (National Center for Biotechnology Information, Bethesda, MD) with the c17-25 EST sequence, and obtained 1,210,178-bp bovine genomic sequence [GenBank:NW_001495576] which contains the *AKIRIN2 *gene and the c17-25 sequence. The bovine *AKIRIN2 *gene spans 22,844 bp and comprises 768 bp of exon 1 including 533 bp of 5' untranslated region (UTR) sequence, 144 bp of exon 2, 150 bp of exon 3, 72 bp of exon 4, 823 bp of exon 5 including 812 bp of 3' UTR sequence, and 4 introns, based on genomic sequence from the NCBI database.

### SNP detection

For single nucleotide polymorphism (SNP) detection, we used two Holstein steers and two somatic nuclear-derived cloned steers [8] from a Japanese Black Itofuku sire with a very high estimated breeding value for marbling [9], which in our previous ddPCR analysis [5] were assigned to low- and high-marbled steer groups, respectively. The two low-marbled Holstein steers and two high-marbled cloned steers were previously shown to have different expression patterns of the c17-25 EST corresponding to a portion of the *AKIRIN2 *gene [5]. The details of DNA samples from these steers were described previously [5]. We used the two high-marbled cloned steers to confirm the authenticity of newly discovered SNP in the *AKIRIN2 *gene.

PCR primers were designed to target ~8-kb proximal promoter and exon regions, to screen polymorphisms in the *AKIRIN2 *gene between two low-marbled Holstein steers and two high-marbled cloned steers. PCR amplification and direct sequencing were performed as described in [10]. Nucleotide polymorphisms were identified by comparison of sequence traces between the 2 steer groups, and designated according to nomenclature for the description of sequence variations in the HGVS (Human Genome Variation Society, Fitzroy, VIC, Australia) [11]. Primer sequences will be available on request.

This sequence analysis revealed only one SNP in the *AKIRIN2 *gene between the 2 steer groups: a G to A transition located 22,220 bp downstream of the transcription initiation site in the 3' UTR (c.*188G>A). The c.*188G>A corresponds to the rs41255597 in the dbSNP [12]. The two Holstein steers were homozygous for the *G *allele at the c.*188G>A site, whereas the two cloned steers homozygous for the *A *allele.

### Association study

#### Samples and data

We performed 3 experiments for association of the c.*188G>A with marbling and subcutaneous fat thickness. We used 100 Japanese Black sires in experiment 1. The sires were or are used in the Oita Prefectural Institute of Animal Industry (Oita, Japan). There was no strong bias for a specific father or a specific maternal grandfather of the sires, and the sire panel likely represents a variety of the sire lines. In experiment 2, 753 paternal half-sib Japanese Black progeny steers (56 to 546 steers per sire) produced from 4 sires heterozygous for the c.*188G>A, with dams considered to represent a random sample of the female population, were used. In experiment 3, we used 730 paternal half-sib Japanese Black progeny steers (59 to 454 steers per sire) produced from 3 sires homozygous for the *A *allele at the c.*188G>A, with dams considered to be a random mating population. The progeny steers in experiments 2 and 3 were fattened and shipped to the carcass market in the Oita prefecture. Semen or blood of the sires and adipose tissues of the progeny steers were collected for SNP genotyping. DNA samples were prepared from the materials according to standard protocols.

The predicted breeding values of the sires and the progeny steers for marbling score and subcutaneous fat thickness were used as phenotypic values in this study. The details of breeding value estimation and scoring methods for marbling and subcutaneous fat thickness were described in [10].

This study conformed to the guidelines for animal experimentation of the Graduate School of Agriculture, Kyoto University (Kyoto, Japan).

#### SNP genotyping

The c.*188G>A was genotyped using PCR-restriction fragment length polymorphism (RFLP) method. PCR primers used for PCR-RFLP were 5'-TCTTAGGCAGCAACCGGATT-3' and 5'-GAAGGGCATGTTCTTAGAATACCAG-3'. Nucleotide positions relative to the transcription initiation site of the *AKIRIN2 *gene were 22,167 to 22,186 and 22,336 to 22,312, respectively. PCR amplifications were carried out as described in SNP detection section, using a final volume of 20 μl and the annealing temperature of 56°C. An aliquot of PCR-amplified products was digested at 37°C for 1 h with restriction enzyme *Fok*I, and electrophoresed on a 3.0% agarose gel. Agarose gels were stained with ethidium bromide and photographed under an ultraviolet light. Using this method, 170-bp PCR fragments containing the SNP site were amplified, and *Fok*I-digested into 65- and 105-bp fragments for the *G *allele, but not for the *A *allele: the *GG *homozygotes, the *AA *homozygotes, and the *GA *heterozygotes resulted in 2 bands (65 and 105 bp), 1 band (170 bp), and 3 bands (65, 105, and 170 bp), respectively.

#### Experiment 1

Genotyping 100 sires for the c.*188G>A revealed 26 animals homozygous *GG*, 48 animals heterozygous *GA *and 26 animals homozygous *AA*. Statistically significant differences among the genotypes of the SNP were detected for marbling (*P *= 0.041), but not for subcutaneous fat thickness (*P *= 0.165), by the analysis with the model that included the SNP genotype as the fixed effect and the sire (father of the sire) as the random effect (Table 1). The marbling score was significantly higher in the *AA *homozygotes than in the *GG *homozygotes, and that of the heterozygotes was intermediate between those of the 2 homozygotes (Table 1).

**Table 1:** Effect of the SNP genotypes on marbling and subcutaneous fat thickness in experiment 1

		Breeding value*	
		
Genotype	No. of animals	MARBLING SCORE	Subcutaneous fat thickness (mm)
*AA*	26	2.96^a ^± 0.33	-3.91 ± 1.11
*GA*	48	2.30^b ^± 0.29	-2.15 ± 0.99
*GG*	26	2.27^b ^± 0.27	-3.19 ± 0.84

*The breeding values are given as estimates ± SE.

^a, b^Estimates at different genotypes without a common letter in their superscripts significantly differ (*P *< 0.05).

#### Experiment 2

To better estimate the effect of genotype of the c.*188G>A on marbling and subcutaneous fat thickness, we used 753 progeny steers from 4 sires heterozygous for the SNP. The interaction between the SNP genotype and the sire was not statistically significant (*P *= 0.544 for marbling, and *P *= 0.260 for subcutaneous fat thickness) in the model that included the SNP genotype and the sire as the fixed effects and their interaction, and was excluded from our statistical model. In the model without the interaction effect, the SNP genotype had the statistically significant effect on marbling (*P *= 0.005), but not subcutaneous fat thickness (*P *= 0.070) (Table 2). Consistent with the result obtained by using the 100 sires, the marbling score was significantly higher in the *AA *homozygotes than in the *GG *homozygotes, and that of the heterozygotes was intermediate between those of the 2 homozygotes (Table 2).

**Table 2:** Effect of the SNP genotypes on marbling and subcutaneous fat thickness in experiment 2

		Breeding value*	
		
Genotype	No. of animals	MARBLING SCORE	Subcutaneous fat thickness (mm)
*AA*	204	2.83^a ^± 0.06	0.13 ± 0.30
*GA*	389	2.75^a, b ^± 0.05	0.47 ± 0.23
*GG*	160	2.54^b ^± 0.07	1.00 ± 0.31

*The breeding values are given as least squares means ± SE.

^a, b^Means at different genotypes without a common letter in their superscripts significantly differ (*P *< 0.01).

#### Experiment 3

To further verify the association of the c.*188G>A with marbling, we used 730 progeny steers from 3 sires homozygous for the *A *allele at the SNP. These steers could be grouped only according to the alleles that they received from their dams, which are considered to be a random sample of a general population in Japanese Black beef cattle. Therefore, this experiment likely allowed a linkage disequilibrium estimate of the effect of the SNP. The interaction between the SNP genotype and the sire was not statistically significant (*P *= 0.513 for marbling, and *P *= 0.732 for subcutaneous fat thickness) in the model that included the SNP genotype and the sire as the fixed effects and their interaction, and was excluded from our statistical model. In the model without the interaction effect, the SNP genotype effect reached statistical significance (*P *= 0.047) for marbling, but not for subcutaneous fat thickness (*P *= 0.455) (Table 3). Genotype profiles within marbling score categories were consistent with the results obtained by experiments 1 and 2 (Table 3).

**Table 3:** Effect of the SNP genotypes on marbling and subcutaneous fat thickness in experiment 3

		Breeding value*	
		
Genotype	No. of animals	Marbling score	Subcutaneous fat thickness (mm)
*AA*	348	3.88^a ^± 0.04	1.39 ± 0.25
*GA*	382	3.74^b ^± 0.03	1.16 ± 0.23

*The breeding values are given as least squares means ± SE.

^a, b^Means at different genotypes without a common letter in their superscripts significantly differ (*P *< 0.05).

Based on 3 experiments, we showed that the c.*188G>A is associated with marbling in Japanese Black beef cattle, with the *A *allele resulting in high levels of marbling. This was especially evident in experiments 2 and 3, because the dams can be considered to represent a random sample of the Japanese Black population and thus the association is likely to be true. Further, the association of the c.*188G>A with marbling was corroborated by an independent study using 104 paternal half-sib Japanese Black families with a total of 821 progeny steers (S. Sukegawa, unpublished data).

Based on the association of the c.*188G>A SNP with marbling, on the expression difference in the c17-25 EST between low- and high-marbled steer groups, and on the co-localization of the marbling QTL with the *AKIRIN2 *gene, we hypothesize that the SNP in the 3' UTR might influence gene expression and marbling by affecting the *AKIRIN2 *mRNA stability. However, a more likely event is that the *AKIRIN2 *SNP is in linkage disequilibrium with an unidentified and truly relevant mutation, rather than functional and a causal mutation for marbling, from a view of biological function that the AKIRIN2 is involved in innate immune responses [13].

The effect of genotypes of the SNP was not statistically significant (*P *> 0.05) for subcutaneous fat thickness. Further, the marbling QTL corresponding to the chromosomal position of the *AKIRIN2 *did not show a statistically significant effect on subcutaneous fat thickness [6]. Thus, it is likely that the *AKIRIN2 *SNP is not associated with subcutaneous fat thickness in Japanese Black beef cattle. This might be supported by the fact that Japanese Black breed exhibits low genetic correlation between marbling and subcutaneous fat thickness [14].

We have recently reported that SNPs in the 5' UTR and the 3' UTR of the *endothelial differentiation*, *sphingolipid G-protein-coupled receptor*, *1 *(*EDG1*) gene [15], and a SNP in the promoter region of the *titin *(*TTN*) gene [10] were associated with marbling in Japanese Black beef cattle. Thus, our present study is an additional report to show polymorphisms associated with marbling using Japanese Black breed.

The information on the *AKIRIN2 *SNP obtained in this study, as well as the *EDG1 *SNPs and *TTN *SNP, may be applied to effective marker-assisted selection to increase the levels of marbling in Japanese Black beef cattle. Further studies will be needed to examine whether the effects of the favourable marbling alleles at each locus are additive, and whether animals homozygous for favourable alleles at the 3 loci have the highest marbling scores.

## Competing interests

The authors declare that they have no competing interests.

## Authors' contributions

TY carried out the statistical analyses and drafted the manuscript. SS, SS, TO, and YT carried out the SNP detection and genotyping. TF, MM, HM, and FM participated in the sample and data collection. TM carried out further statistical analyses. YS participated in the design and coordination of the study and helped to draft the manuscript. All authors read and approved the final manuscript.
